# Synergistic Mechanism of Hydroxyl Regulation and a Polyvinylpyrrolidone Surfactant in Enhancing the Catalytic Oxidation Abilities of BiOBr

**DOI:** 10.3390/molecules30061286

**Published:** 2025-03-13

**Authors:** Yiran Zhang, Boyuan Xuan, Jiekai Wang, Xiang Chen, Changwei Zhao, Lixia Zhao, Jing Kang

**Affiliations:** 1 Beijing Key Laboratory of Farmland Soil Pollution Prevention and Remediation, College of Resources and Environmental Sciences, China Agricultural University, Beijing 100193, China; zhangyrann@163.com (Y.Z.); m15901333686@163.com (B.X.); wjiekai@126.com (J.W.); 2 State Key Laboratory of Water Environment Simulation, School of Environment, Beijing Normal University, Beijing 100875, China; chenxiang@mail.bnu.edu.cn; 3 State Key Laboratory of Environmental Chemistry and Ec-Toxicology, Research Center for Eco-Envronmental Sciences, Chinese Academy of Sciences, Beijing 100085, China; zlx@rcees.ac.cn; 4 China Institute for Radiation Protection, Taiyuan 030006, China; rabbit0766@163.com

**Keywords:** photocatalysis, BiOBr, hydroxyl, surfactant, photooxidative ability, ciprofloxacin, dyes

## Abstract

The rational design of BiOBr photocatalysts with optimized surface properties and enhanced photooxidative capacities is crucial. This study proposes a synergistic strategy combining hydroxyl-rich solvents with polyvinylpyrrolidone (PVP) surfactants to modulate the structural and electronic properties of BiOBr through a solvothermal approach. The resulting self-assembled microspheres demonstrated exceptional efficiency in degrading ciprofloxacin (CIP), methyl orange (MO), and rhodamine B (RhB). Among the synthesized variants, BiOBr-EG-PVP (fabricated with ethylene glycol and PVP) exhibited the highest photocatalytic activity, achieving near-complete removal of 20 mg/L CIP and RhB within 10 min under visible light irradiation, with degradation rates 60.12–101.73 times higher than pristine BiOBr. The structural characterization revealed that ethylene glycol (EG) not only induced the formation of self-assembled microspheres but also introduced abundant surface hydroxyl groups, which simultaneously enhanced the hole-mediated oxidation capabilities. The incorporation of PVP further promoted the development of hierarchical honeycomb-like microspheres and synergistically enhanced both the hydroxyl group density and photooxidative potential through interfacial engineering. Density functional theory (DFT) calculations confirmed that the enhanced photooxidative performance originated from an increased surface oxygen content. This work elucidates the synergistic effects of hydroxyl-rich solvents and surfactant modification in the fabrication of advanced BiOBr-based photocatalysts, providing new insights for high-performance photocatalysis for environmental remediation.

## 1. Introduction

An increasing amount of organic wastewater, such as antibiotic-containing and dyeing wastewaters, is resistant to treatment, leading to serious environmental hazards [1,2]. By 2030, the world’s veterinary antibiotic consumption is predicted to reach 107,472 tons [3], with ciprofloxacin (CIP) being widely used in humans and one of the most frequently prescribed fluoroquinolones in veterinary medicine. Currently, the direct discharge and inadequate removal of CIP causes over half of the consumed CIP to be released into various environmental settings [4,5]. This indiscriminate discharge can result in secondary pollution, such as the spread of genes that cause antibiotic resistance, which raises environmental risks and healthcare expenses [6]. Furthermore, it is reported that over 700,000 tons of dyes are produced annually, with about 30% of that amount being released into the environment [7]. Wastewater that contains dyes that do not readily biodegrade can harm aquatic ecosystems by impeding vital functions like photosynthesis, which, in turn, affects how well aquatic life functions [8]. Nowadays, adsorption, membranes, sophisticated oxidation processes, and other techniques are frequently employed for the treatment of such water [9,10,11]. Among them, photocatalytic technology, as a clean and efficient method, has received widespread attention from researchers [12]. BiOBr is a typical visible-light-responsive photocatalyst with a unique layered structure (composed of (Bi_2_O_2_)^2+^ and Br^−^), controllable bandgap energy, and appropriate valence band (VB) potential, which has been widely used in the photocatalytic degradation of organic pollutants [13,14,15,16,17]. However, the undesirable morphology and size, and unsuitable energetic band structure of pristine BiOBr limit its applicability.

Surface modification of a catalyst is an effective method to improve its photocatalytic activity [18,19]. Many studies have utilized solvent molecules to regulate the morphology and surface atomic structure of photocatalytic materials during material synthesis [19,20]. Previous research used the hydroxyl-rich surface strategy to modify the surface of catalysts and enhance the number of active sites, since hydroxyl radicals are crucial intermediates in photocatalytic oxidation reactions and play an important role in the adsorption of organic pollutants [21,22,23]. Cui et al. (2022) demonstrated that the formation of hydrogen bonds between surface hydroxyl groups and organic pollutants significantly enhances the adsorption capacity of Bi_4_O_5_I_2_ [24]. Wang et al. (2021) employed mesoerythritol, a polyhydroxy compound, to engineer the surface of BiOBr nanosheets, resulting in enhanced oxidative activity that achieved rapid degradation of phenol and rhodamine B (RhB) within 30 min [25]. However, the question of how the commonly used solvents with hydroxyl groups affect the structure–activity relationship of BiOBr needs to be explored.

Furthermore, a catalyst’s morphology significantly impacts its adsorption performance and the diffusion resistance of the photogenerated electrons [26]. Adjustments of the reaction parameters, including the solution composition, reaction time, temperature, and addition of surfactants, have been widely used in the regulation of catalyst morphologies [27,28,29,30]. Polyvinyl pyrrolidone (PVP) is a capping agent that has aroused significant attention in the synthesis of catalysts due to its non-toxicity, high stability, bio-compatibility, and steric effects [31,32]. Due to its advantageous qualities, such as molecular inclusion capabilities, chiral modification potential, and efficient charge carrier transfer abilities, PVP is a viable candidate material for modifying nanocrystals to enhance their catalytic performance [33,34,35,36]. Zhang et al. (2020) demonstrated that loading PVP could reduce the BiOBr particle size and increase its specific surface area [34]. This could potentially create more channels and contact area, thereby promoting more intimate interactions between pollutants and reactive sites. More research is needed to fully understand the combined impact of solvent hydroxyl control and the addition of PVP in the hydrothermal synthesis process of BiOBr.

Various hydroxyl-rich alcohols (mannitol (Ma), ethylene glycol (EG), glycol (Gl), and methanol (Me)) were applied in this work to examine the impact of hydroxyl-rich solvents on the composition and characteristics of BiOBr. To investigate the synergistic impact of the two modification techniques mentioned above, the surfactant PVP (K30) was used. The hydroxyl-rich solvent effectively raised the BiOBr material’s surface hydroxyl content and improved CIP, rhodamine B (RhB), and methyl orange (MO) adsorption. Additionally, it raised BiOBr’s valence band (VB), which greatly improved its capacity to oxidize ciprofloxacin and organic dyes under visible light. The addition of PVP regulated the synthesis of self-assembled microspheres, increased the specific surface area and pore volume of BiOBr, and further enhanced the oxidation capability of BiOBr. The prepared BiOBr-based catalysts exhibited the desirable stability and catalytic performance under solar irradiation, making them potential candidates for wastewater treatment applications.

## 2. Results and Discussion

### 2.1. Morphology and Structure of Photocatalysts

**Figure 1:**
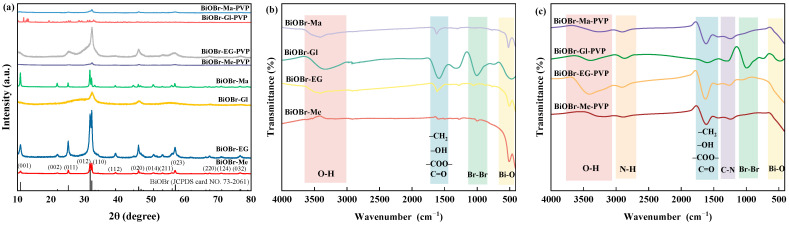
(**a**) XRD patterns of BiOBr-Me, BiOBr-EG, BiOBr-Gl, BiOBr-Ma, BiOBr-Me-PVP, BiOBr-EG-PVP, BiOBr-Gl-PVP, BiOBr-Ma-PVP, (**b**) FT-IR spectra of BiOBr-Me, BiOBr-EG, BiOBr-Gl, BiOBr-Ma, and (**c**) BiOBr-Me-PVP, BiOBr-EG-PVP, BiOBr-Gl-PVP, and BiOBr-Ma-PVP.

The crystalline structures of various BiOBr-based catalysts were characterized by X-ray diffraction (XRD), as shown in Figure 1a. The diffraction patterns exhibited characteristic peaks of tetragonal BiOBr at 10.95°, 21.99°, 25.26°, 31.81°, 32.31°, 39.43°, 46.35°, 50.84°, 53.51°, 58.23°, 67.63°, 71.23°, and 76.68°, all of which were impurity-free and matched well with the reference card (JCPDS No. 73–2061) [37]. Notably, the diffraction peak widths of samples prepared in glycerol solution were broader compared to those synthesized in other solvents, suggesting reduced crystallinity. This observation could be attributed to the high boiling point and strong adsorption characteristics of glycerol, which may have led to molecular residues being left on the BiOBr surface or their incorporation into lattice interstices, thereby inducing surface disorder and local structural distortions [23]. The addition of PVP as a capping agent further reduced the crystallinity of the catalysts, primarily due to its surface shielding effects. Increasing the amount of PVP (BOB-0.4, BOB-0.6, and BOB-0.8) did not obviously alter the exposed crystal planes or crystallinity (Figure S1), consistent with the results of a previous study [34]. In addition, the sustained expansion of the vertices of the (110) plane suggested preferential crystal growth along the (110) direction, consistent with the role of PVP in promoting the exposure of the (110) plane [28]. Meanwhile, the (001) crystal plane and adjacent (012) crystal plane were suppressed due to their low surface energy and competitiveness with other planes.

Fourier-transform infrared (FT-IR) spectroscopy was systematically performed to elucidate the structural characteristics of the synthesized catalysts. As shown in Figure 1b,c, the characteristic peaks at approximately 520 cm^−1^ were assigned to the Bi-O stretching mode, and the bands in the range of 830–1150 cm^−1^ were attributed to Br-Br [38,39], confirming the successful formation of BiOBr. The adsorption peaks observed at 1400–1800 cm^−1^ were identified as vibrations of -CH_2_, -OH, -COO-, and C=O groups [34], and the broad peaks at 3000–3650 cm^−1^ were associated with the O-H stretching motion of adsorbed water molecules [40]. Notably, BiOBr-Gl exhibited more intense peaks corresponding to O-H and C-O functional groups, providing strong evidence of residual glycerol molecules on the BiOBr surface. Additionally, new characteristic peaks appeared at 1150–1400 cm^−1^ and 2700–3000 cm^−1^, which were attributed to the tensile vibration of C-N heterocyclic rings in PVP and the stretching motions of N-H and O-H bonds in PVP and absorbed water molecules [41]. These spectroscopic features provided compelling evidence for the successful integration of PVP with BiOBr, confirming the effective modification of the catalyst surface.

**Figure 2:**
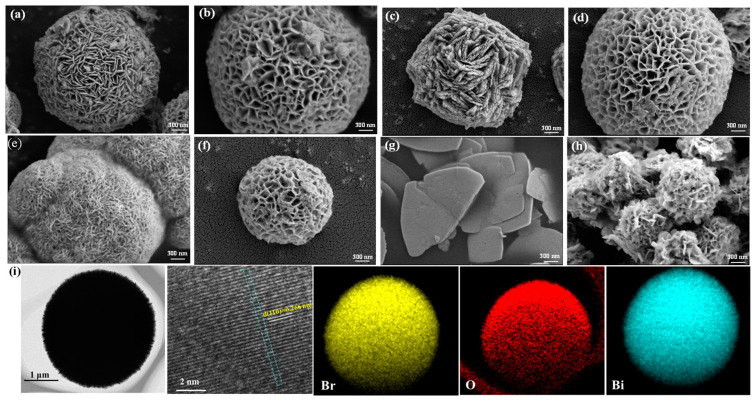
SEM images of (**a**) BiOBr-Me, (**b**) BiOBr-Me-PVP, (**c**) BiOBr-EG, (**d**) BiOBr-EG-PVP, (**e**) BiOBr-Gl, (**f**) BiOBr-Gl-PVP, (**g**) BiOBr-Ma, (**h**) BiOBr-Ma-PVP, and TEM images of (**i**) BiOBr-EG-PVP.

The morphological evolution and microstructural characteristics of the synthesized catalysts were comprehensively investigated using scanning electron microscopy (SEM) and transmission electron microscopy (TEM). As shown in Figure 2a–h, the morphologies varied significantly depending on the solvents and PVP used during synthesis. The average size of all catalysts ranged from 1 to 4 μm. BiOBr-Me exhibited sheet-like self-assembled microspheres. BiOBr-EG exhibited a more pronounced stacking of sheet-like self-assembled microspheres, while BiOBr-EG displayed more pronounced stacking of similar sheet-like microspheres, along with better dispersion, which was attributed to the viscosity and polarity of ethylene glycol (Figure S2). In contrast, BiOBr-Gl formed a unique compact structure with reduced interparticle spacing and increased aggregation, resulting from the high viscosity of glycerol that inhibited particle development. BiOBr-Ma adopted a flake-like morphology, likely due to the presence of mannitol promoting faster crystal growth along specific crystallographic orientations (e.g., the (001) plane), consistent with the enhanced intensity of the (001) diffraction peak in the corresponding XRD pattern.

Notably, the introduction of PVP induced significant morphological transformations. BiOBr-Me-PVP transformed into honeycomb-shaped microspheres. The main reason for this was PVP restricting the reaction rate and thinning the nanosheets that constitute the microspheres [33]. BiOBr-EG-PVP exhibited similar but more uniformly dispersed morphologies to BiOBr-Me-PVP (Figure S4). Similarly, the addition of PVP enable BiOBr-Gl-PVP to retain the microstructure and morphology of BiOBr-Me-PVP and BiOBr-EG-PVP. In the case of BiOBr-Ma-PVP, PVP facilitated the formation of spherical structures, as further confirmed by the morphological changes of BOB-W and BOB-P (Figure S3a,b). Brunauer-Emmett-Teller (BET) surface area analysis (Table S1) revealed that BiOBr-EG-PVP possessed a larger specific surface area and a richer pore structure, consistent with its well-dispersed, non-agglomerated morphology (Figure S2).

TEM investigation was performed using BiOBr-EG-PVP as a representative prototype to further examine the microstructure of BiOBr-based catalysts due to its optimal morphology (Figure 2f). The structure of BiOBr-EG-PVP was made up of microspheres that had a diameter of roughly 3.30 μm. The highly reactive single crystal (110) facet matched the continuous interplanar lattice fringes of 0.266 nm [42]. Bi, O, and Br were consistently dispersed, as revealed by the EDS elemental map, which was compatible with the successful in situ synthesis of BiOBr.

**Figure 3:**
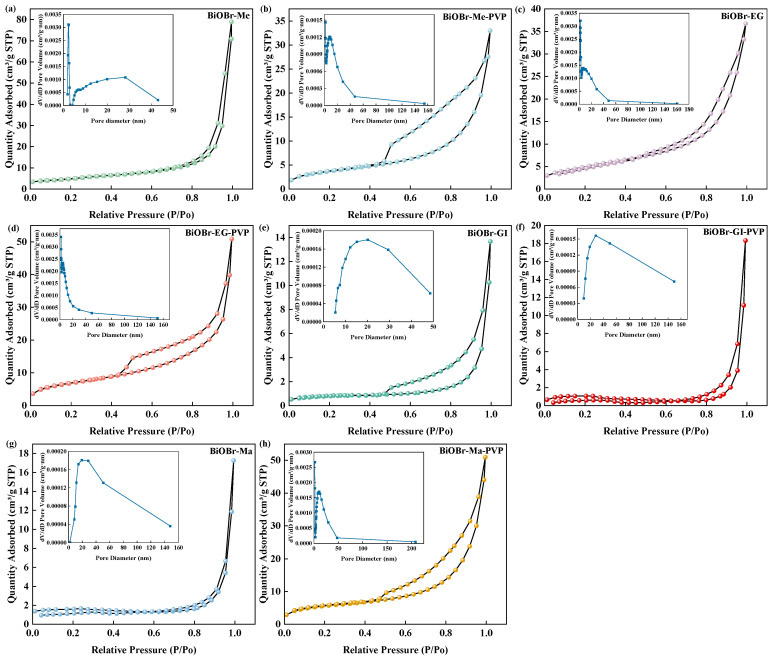
N_2_ adsorption/desorption isotherm and pore size distribution of (**a**) BiOBr-Me, (**b**) BiOBr-Me-PVP, (**c**) BiOBr-EG, (**d**) BiOBr-EG-PVP, (**e**) BiOBr-Gl, (**f**) BiOBr-Gl-PVP, (**g**) BiOBr-Ma, and (**h**) BiOBr-Ma-PVP.

All synthetic BiOBr catalysts exhibited a type IV nitrogen adsorption–desorption isotherm with a typical H3 hysteresis loop that, as shown in Figure 3a–h, confirmed the presence of a mesoporous structure [43]. The specific surface areas of the catalysts were calculated using the Brunauer-Emmett-Teller (BET) method (Table S1). Among the photocatalysts synthesized from the four alcoholic solutions, BiOBr-Me and BiOBr-EG demonstrated significantly higher specific surface area and pore volume, further supporting the superior structural properties of their porous microsphere formations. However, the addition of PVP reduced the specific surface area of BiOBr-Me-PVP to 13.28 m^2^/g from the predicted 18.05 m^2^/g of BiOBr-Me, primarily due to the shrinkage of the macropore size and total pore volume. The specific surface area of BiOBr-EG increased by 1.35 times compared to BiOBr-EG (18.38 m^2^/g), reaching 24.87 m^2^/g, the largest among all the prepared BiOBr based catalysts. Larger pore volumes and specific surface areas can provide greater contact area and more active sites [44]. However, excessive PVP addition beyond the optimal concentration led to a reduction in both pore volume and specific surface area, likely due to structural deformation caused by PVP overloading (Figure S4). Among the synthesized catalysts, BiOBr-Gl and BiOBr-Gl-PVP exhibited the lowest specific surface areas of 3.05 m^2^/g and 4.15 m^2^/g, respectively, consistent with their low crystallinity characteristics. Notably, the addition of PVP greatly increased the specific surface area of BiOBr-Ma-PVP, which reached 20.70 m^2^/g, i.e., 3.36 times that of BiOBr-Ma; this was primarily ascribed to the formation of self-assembled microspheres, which reduced the pore size and increased the overall pore volume. Overall, PVP addition enhanced the micropore volume, consequently enhancing the total pore volume and specific surface area. Additionally, as the hydroxyl content in the solvent increased, the specific surface area of the catalysts initially increased and then declined. 

**Figure 4:**
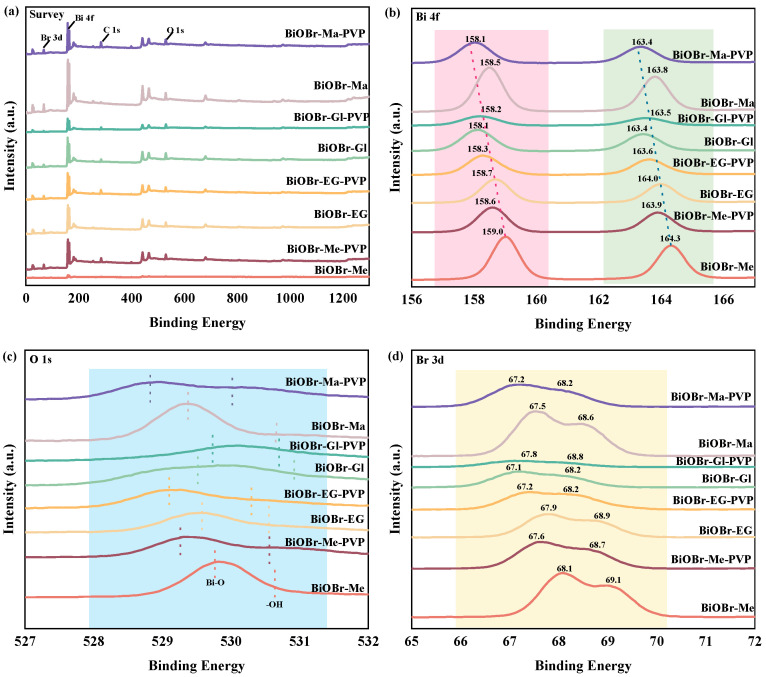
XPS spectra of BiOBr-Me, BiOBr-EG, BiOBr-Gl, BiOBr-Ma, BiOBr-Me-PVP, BiOBr-EG-PVP, BiOBr-Gl-PVP, BiOBr-Ma-PVP (**a**) Survey spectrum, (**b**) Bi 4f, (**c**) O 1s, (**d**) Br 3d.

X-ray photoelectron spectroscopy (XPS) analysis was employed to investigate the surface chemical composition and electronic states of the synthesized BiOBr-based catalysts. The binding energy peaks observed at 68 eV (Br 3d), 158 eV (Bi 4f), 285 eV (C 1s), and 530 eV (O 1s) confirmed the predominant composition of the catalysts (Figure 4a–d). In the high-resolution Bi 4f spectra, two peaks at 158.1–159.0 eV and 163.4–164.3 eV were assigned to Bi 4f_7/2_ and Bi 4f_5/2_, respectively. The O 1s spectra were deconvoluted into two peaks at 530–530.8 eV and 528.8–529.8 eV, corresponding to surface oxygen in hydroxyl groups and Bi-O bonds within the BiOBr layered structure [45,46,47]. A comparison of the hydroxyl and lattice oxygen contents via XPS further elucidated the effects of different solvents and PVP addition levels. As shown in Table S2, all oxygen species types in BiOBr synthesized from deionized water were lattice oxygen. However, the use of hydroxyl-rich solvents increased the amount of hydroxyl groups (Figure S5), which was advantageous for the adsorption and degradation of organic contaminants [24]. Additionally, the introduction of PVP considerably increased the hydroxyl group contents (Table S2), indicating a synergistic effect between PVP and hydroxyl-rich solvents. The Br 3d spectra were fitted into two peaks, Br 3d_5/2_ and Br 3d_3/2_, located at 67.1–68.1 eV and 68.2–69.1 eV, respectively. The binding energies of Bi 4f, O 1s, and Br 3d_3/2_ shifted to lower energies as the hydroxyl content of the solvent increased, a trend that was further enhanced by the addition of PVP. This shift suggested electronic interaction between BiOBr and PVP components [22].

### 2.2. Photocatalytic Performance of Degradation for Organics

Extensive studies have demonstrated that heterogeneous photocatalysts exhibit an optimal dosage for achieving maximum photocatalytic efficiency [48,49]. As illustrated in Figure S6 and Table S3, the photocatalytic performance of BiOBr-EG-PVP toward RhB degradation showed a strong dependence on catalyst loading. The photodegradation rate increased from 0.11816 min^−1^ to 0.28460 min^−1^ as the BiOBr-EG-PVP dosage rose from 0.40 g/L to 1.0 g/L. Nevertheless, a further increase to 1.20 g/L and 1.40 g/L resulted in a slight decline in the photodegradation rate. This phenomenon could be attributed to the formation of excessive nanoparticle layers at higher concentrations (>50 mg), which caused light scattering and screening effects, thereby reducing photon availability and consequently diminishing photocatalytic activity [50]. Based on these findings, the optimal catalyst dosage for subsequent photocatalytic experiments was determined to be 1.0 g/L.

**Figure 5:**
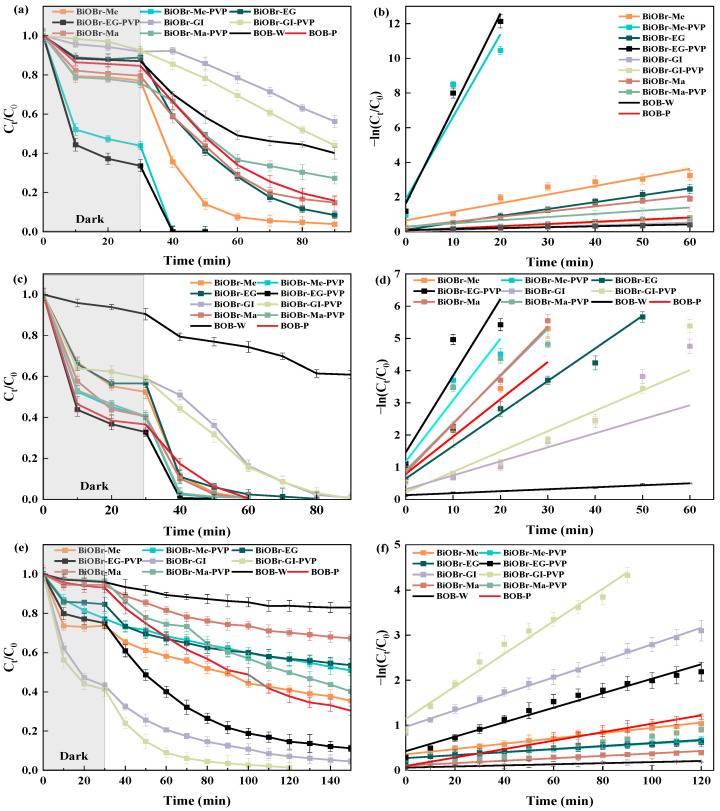
(**a**) Photodegradation of CIP with all catalysts under visible light, (**b**) first-order-kinetic plots and corresponding apparent rate constants k of all catalysts of CIP photodegradation, (**c**) photodegradation of RhB with all catalysts under visible light, (**d**) first-order-kinetic plots and corresponding apparent rate constants k of all catalysts of RhB photodegradation, (**e**) photodegradation of MO with all catalysts under visible light, and (**f**) first-order-kinetic plots and corresponding apparent rate constants k of all catalysts of MO photodegradation.

The photocatalytic performance of the synthesized catalysts was systematically evaluated through the degradation of ciprofloxacin (CIP), rhodamine B (RhB), and methyl orange (MO) under visible light irradiation. As shown in Figure 5a–f, all catalysts basically achieved adsorption equilibrium within 30 min, and extending the dark reaction time further did not significantly affect the adsorption efficiency. Overall, the inclusion of PVP restricted the size of BiOBr microspheres, leading to a larger specific surface area, smaller pore size, and higher -OH quantity, all of which contributed to improved adsorption capacity [34]. Notably, the addition of PVP significantly enhanced the adsorption capacity of BiOBr-EG-PVP for CIP and RhB, while BiOBr-Gl-PVP demonstrated superior adsorption of MO. 

After achieving adsorption–desorption equilibrium, the photocatalytic oxidation process was initiated. BiOBr-Me-PVP and BiOBr-EG-PVP achieved approximately 100% removal of CIP within 10 min (Figure 5a–f), while BiOBr-Me and BiOBr-EG degraded over 90% of CIP within 60 min. This high efficiency could be attributed to the ample active sites provided by their large specific surface areas. Similar outcomes were observed for RhB degradation. BiOBr-Me-PVP and BiOBr-EG-PVP degraded approximately 100% of RhB within 10 min, whereas BiOBr-Me, BiOBr-Ma, and BiOBr-Ma-PVP required 30 min. In contrast, BiOBr prepared using glycerol as a solvent exhibited superior degradation performance for MO, primarily due to its enhanced adsorption properties. Overall, BiOBr-EG-PVP demonstrated the optimum photocatalytic degradation capability, owing to the larger specific area and -OH amount. The catalytic reaction followed the pseudo-first-order kinetic equation, i.e., the corresponding reaction equations as described by Wang et al. (2015) [51]. A quantitative analysis revealed that BiOBr-EG-PVP exhibited the fastest CIP and RhB degradation rate (*k* = 0.54732 min^−1^ and 0.28460 min^−1^, respectively) (Table S4). The degradation efficiency and apparent rate reported in the research were higher than those in most literature reports [52,53,54], demonstrating the superior photocatalytic performance of the developed catalysts.

**Figure 6:**
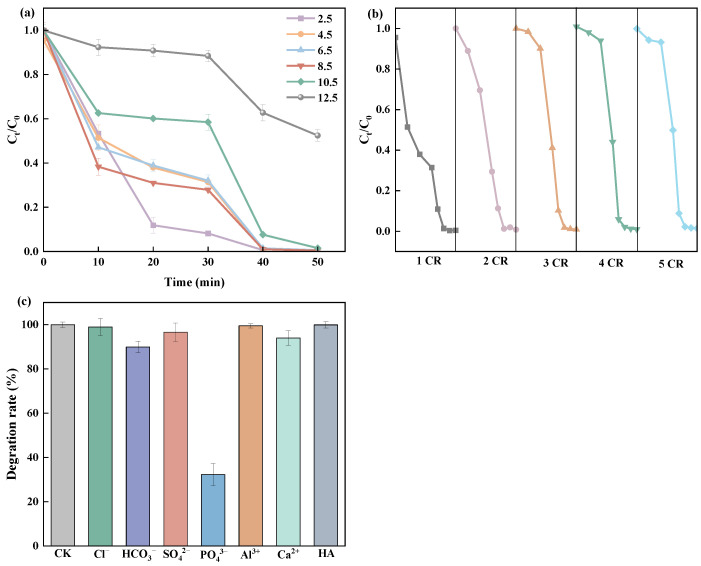
(**a**) Initial pH value on BiOBr-EG-PVP catalytic degradation of RHB, (**b**) cyclic stability of BiOBr-EG-PVP by the removal of RhB, and (**c**) coexistence of cations, anions, and organic pollutants on the removal efficiency of BiOBr-EG-PVP.

The BiOBr-EG-PVP catalyst demonstrated exceptional cycle stability and remarkable resistance to pH variations, as demonstrated in Figure 6a,b. Under visible light irradiation, BiOBr-EG-PVP maintained approximately 100% of the RhB degradation rate within 15–20 min across a pH range of 2.5–10.5. The degradation rate significantly decreased to 47.6% at pH 12.5, primarily due to the strong alkaline conditions impairing BiOBr-EG-PVP’s ability to adsorb RhB, thereby hindering the catalytic degradation process [55]. To evaluate structural stability under varying pH conditions, post-cycling XRD characterization was conducted on samples spanning the pH range of 2.5–10.5 (Figure S7). The results demonstrated that the crystal structure of all samples remained largely unaffected, indicating robust structural integrity under varying pH conditions. 

Figure 6b displays the cyclic stability of BiOBr-EG-PVP. Samples from the optimal dosage tests were collected to ensure a consistent dosage of 1.0 g/L for each cycle. After each photocatalytic reaction, the catalyst was collected, centrifuged, filtered, and thoroughly washed, followed by drying at 60°C for subsequent reuse. Remarkably, the catalyst maintained consistent photocatalytic performance through five consecutive cycles, demonstrating excellent recyclability. Although a significant decrease in adsorption capacity was observed after the first cycle, attributed to approaching adsorption saturation, this did not compromise the photocatalytic activity, further confirming the catalyst’s stability and practical applicability. 

The influence of common inorganic ions on photocatalytic performance was investigated. As shown in Figure 6c, no obvious inhibition effects could be observed with the addition of these inorganic ions (Cl^−^, NO_3_^−^, HCO_3_^−^, SO_4_^2−^, Al^3+^, and Ca^2+^), except with the addition of PO_4_^3−^ ions. The inhibition of PO_4_^3−^ contributed to its competition with RhB for adsorption on the surface of BiOBr microspheres to form the BiPO_4_, and its stronger electronegativity suppressed the effect of h^+^ [56]. In addition, the presence of HA did not affect RhB removal efficiency, further proving the robust stability of BiOBr-EG-PVP in complex aqueous environments.

### 2.3. Photocatalytic Mechanism 

**Figure 7:**
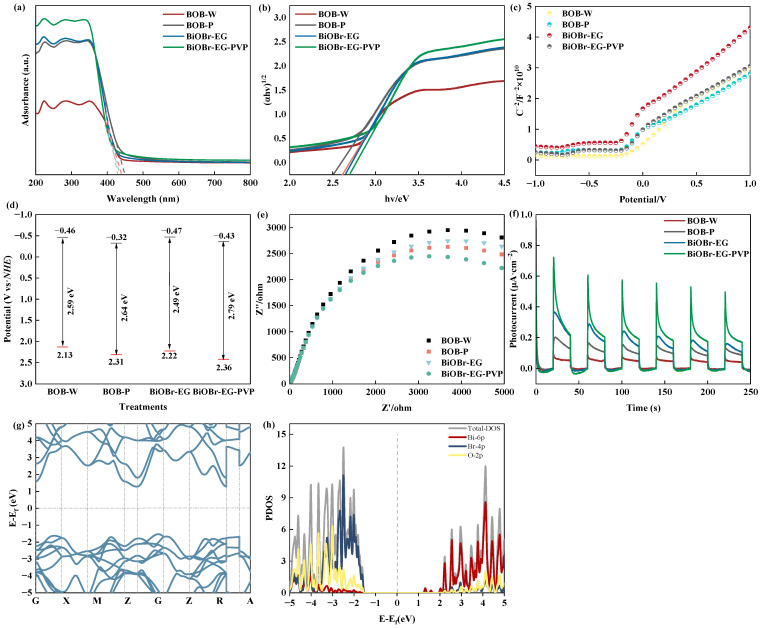
(**a**) UV-vis DRS, (**b**) Tauc plots for the band gap energies originated from UV-Vis DRS, (**c**) Mott-Schottky plots, (**d**) schematic bandgap structures, (**e**) electrochemical impedance spectra, (**f**) photocurrent response of BOB-W, BOB-P, BiOBr-EG, and BiOBr-EG-PVP, (**g**) calculated band structure of BiOBr-EG-PVP, and (**h**) DOS of BiOBr-EG-PVP.

Given the enhanced performance of BiOBr prepared with ethylene glycol and PVP, we explored the synergistic effects of these components using BOB-W and BOB-P as controls. The light absorption properties of the BiOBr-based photocatalytic materials were characterized by UV-vis diffuse reflectance spectroscopy (DRS) (Figure 7a). The absorption edges of BiOBr-EG and BiOBr-EG-PVP were roughly 440 nm with no discernible variation to control treatments (BOB-W and BOB-W). However, BiOBr-EG-PVP exhibited significantly enhanced light absorption intensity across the visible spectrum compared to other catalysts, correlating well with its superior organic pollutant degradation efficiency. These results suggested that while the visible light absorption boundary remained unchanged, the improved light harvesting capability of BiOBr-EG-PVP contributed to its enhanced photocatalytic performance.

The optical band gap energies of the synthesized catalysts were determined using the Tauc plot method based on the equation (αhν) = A(hν − Eg)^n/2^, where α, h, ν, A, and Eg represent the absorption coefficient, Planck constant, light frequency, proportionality constant, and band gap energy, respectively. Additionally, the band gap of the prepared catalysts was calculated using the formula: ahν = A(hν − Eg)^n/2^ (a, h, v, A and Eg represent the absorption coefficient, planck constant, light frequency, and the band gap, respectively) [57,58]. The plots (αhν)^1/2^ versus the photon energy (hν) are displayed in Figure 7b, with the band gap energy values estimated by extrapolating the linear portion of the spectrum to the intersection with the photon energy (hν) axis. 

The flat band potential was evaluated through Mott-Schottky analysis, where the tangent line was drawn along the most linear portion of the M-S curve, and the corresponding potential value was determined from its intercept with the *x* axis. As shown in Figure 7c, the flat band potential for each catalyst was predicted to range between −0.35 and −0.16 eV. The band gap energies (Eg) are summarized in Figure 7d. The conduction band (CB) position was within the range of −0.47 to −0.32 eV, which enabled the photogenerated electrons (e^−^) to directly reduce dissolved oxygen (O_2_/•O_2_^−^ = −0.33 eV) to form superoxide radicals (•O_2_^−^) and hydroxyl radicals (•OH). The valence band (VB) positions ranged from 2.02 to 2.36 eV, with the addition of PVP leading to an increase in the VB potential. The VB position determined the oxidation ability of photocatalytic catalysts, as it governed the generation of highly oxidative holes (h^+^) under light irradiation. The enhanced oxidation ability aligned with the dominant role of h^+^ in the photocatalytic degradation process observed in this study.

Electrochemical experiments were used to evaluate the separation efficiency of photo-charges (e^−^-h^+^). Electrochemical impedance spectroscopy (EIS) analysis revealed that BiOBr-EG-PVP exhibited the smallest arc radius in the Nyquist plot (Figure 7e), indicating that it possessed the lowest charge transfer resistance and consequently the highest charge transport efficiency among the investigated samples [59]. This enhanced performance might be attributed to the synergistic effects of ethylene glycol and PVP, with PVP playing a more dominant role in improving mass transfer efficiency through its structural regulation function. 

To further evaluate the photogenerated charge carrier transfer ability, transient photocurrent responses were measured under visible light irradiation (λ > 420 nm) (Figure 7f). Consistent with its superior photocatalytic performance in pollutant degradation, BiOBr-EG-PVP demonstrated the highest photocurrent intensity, which correlated with its well-developed porous structure and large specific surface area. These structural advantages, achieved through the PVP-assisted synthesis, facilitated efficient charge separation and transfer, ultimately contributing to the enhanced photocatalytic activity.

**Figure 8:**
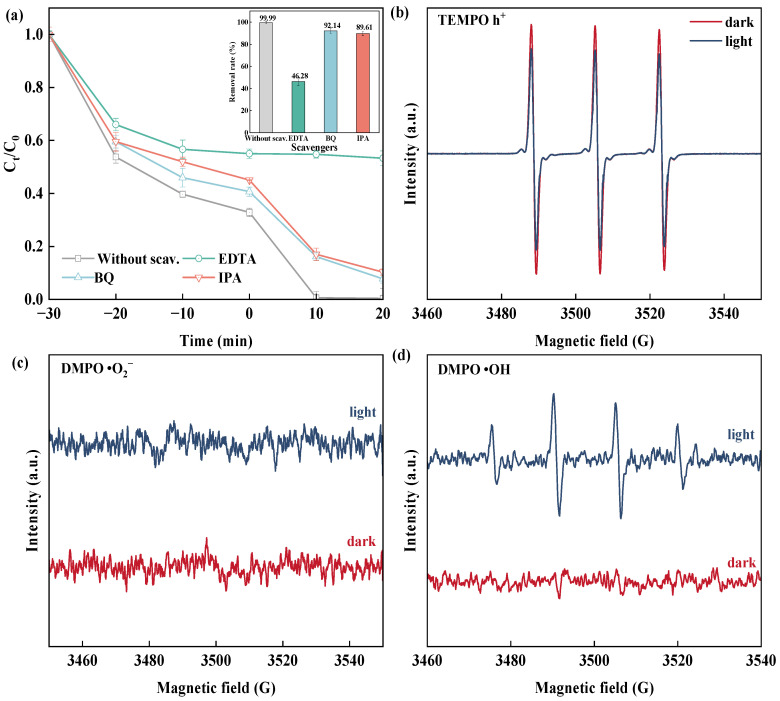
(**a**) The effects of different quenchers on the BiOBr-EG-PVP degradation of RhB, (**b**) ESR spectra from TEMPO h^+^ of BiOBr-EG-PVP, (**c**) ESR spectra from DMPO •O_2_^−^ of BiOBr-EG-PVP, and (**d**) ESR spectra for DMPO •OH of BiOBr-EG-PVP.

DFT calculation was employed to further examine the band structure. As shown in Figure 7g,h, BiOBr-EG-PVP was identified as an indirect semiconductor; this was beneficial to the separation of photogenerated charge carriers. The calculated Eg value of BiOBr-EG-PVP was 2.81 eV, consistent with the experimental result. According to density of states (DOS) calculation (Figure 8b), the valence band maximum (VBM) of the BiOBr-EG-PVP was primarily composed of Br 4p and O 2p orbitals, while the conduction band minimum (CBM) consisted of Bi 6p and O 2p orbitals. Both the theoretical calculations and XPS results indicated that the increased VB potential was mainly attributed to the higher O element content, which enhanced the photooxidative ability of the catalyst.

To identify the active species involved in the photocatalytic process, comprehensive quenching experiments and electron spin resonance (EPR) analyses were conducted. P-benzoquinone (p-BQ), isopropanol (IPA), and disodium ethylenediaminetetraacetate (EDTA-2Na) were applied to the catalytic system as scavengers of •O_2_^−^, •OH, and h^+^, respectively. It was clear from the addition of EDTA-2Na that the BiOBr-EG-PVP catalytic reaction was suppressed, and the addition of IPA and BQ hardly affected the catalytic reduction of RhB (Figure 8a). These results indicated that h^+^ played a pivotal role in the catalytic process, whereas •OH and •O_2_^−^ were not the predominant active species responsible for RhB degradation.

The generation and evolution of radical species during the photocatalytic process were further investigated using EPR spectroscopy coupled with spin trapping techniques. Specifically, DMPO (5,5-dimethyl-1-pyrroline N-oxide) and TEMPO (2,2,6,6-tetramethylpiperidine-1-oxy) were used as spin capture agents in aqueous and methanol solutions, respectively, to capture and detect free radical species (Figure 8b,c). All catalysts revealed significant h^+^ signals, which weakened considerably after 10 min of illumination, further verifying the dominant role of h^+^ in RhB reduction [60]. Additionally, no •O_2_^−^ signals were detected, while weak •OH signals were observed. This phenomenon could be attributed to the reaction of H_2_O with h^+^, leading to the generation of •OH and resulting in the observed weak signals (Figure 8d).

### 2.4. Analysis of Degradation Pathway

**Figure 9:**
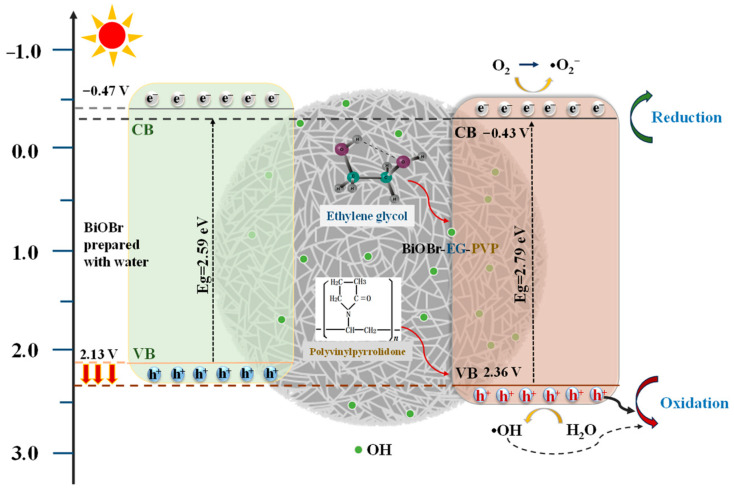
The proposed reaction pathway for organic pollutants degradation by BiOBr-EG-PVP.

Based on the above experimental and theoretical calculation results, a possible mechanism of organic pollutant reduction was proposed and is exhibited in Figure 9. The use of alcohol solvents successfully increased the content of hydroxyl groups on the surface of BiOBr-based photocatalysts and adjusted the sheet-like structure of the material to a stacked spherical shape. Furthermore, the addition of PVP facilitated the formation of a more uniform honeycomb spherical self-assembled structure, providing the material with additional active sites. This modification also increased the proportion of O elements, which contributed to a higher VB potential, thereby enhancing the oxidation ability of h^+^. Given the high VB potential (>1.99 eV), the degradation of organic pollutants was mainly mediated by h^+^, as described in Equations (1)–(4).

BiOBr + hν → e^−^ + h^+^ (1)

H_2_O + h^+^ → •OH + h^+^ (2)

h^+^ + Organic molecule → degraded products (3)

•OH + Organic molecule → degraded products(4)

## 3. Materials and Methods

### 3.1. Chemicals and Materials

All reagents used in this study were analytical grade and employed without further purification. Deionized (DI) water (18.2 kΩ) was used throughout this research. Bismuth nitrate pentahydrate (BiNO_3_·5H_2_O, 99.0%), potassium bromide (KBr, 99.0%), methanol (CH_4_O), ethylene glycol (C_2_H_6_O_2_), glycerol (C_3_H_8_O_3_), mannitol (C_6_H_14_O6), CIP, RhB, MO, ethylenediaminetetraacetic acid disodium salt (EDTA), and p-benzoquinone (p-BQ) were purchased from Aladdin CO. (Shanghai, China). Polyvinylpyrrolidone (PVP (K30)), NaCl, NaHCO_3_, Na_2_SO_4_, Na_3_PO_4_, Al(NO)_3_, and CaCl_2_, humic acid (HA), and isopropyl alcohol (IPA) were supplied from Macklin Biochemical Co. (Shanghai, China).

### 3.2. Preparation of Photocatalysts

The BiOBr and BiOBr-PVP samples were synthesized using a facile solvothermal method. Firstly, 1 mmol (Bi(NO_3_)_3_·5H_2_O, 99.0%) and a specified amount of PVP (0 g, 0.2 g) were magnetically stirred for 30 min at room temperature in a 60 mL alcohol solution. The alcohol solutions used included methanol, ethylene glycol, glycerol, and mannitol. Among them, the amount of mannitol added was 0.1 mmol. Then, 1 mmol KBr was added to the mixture and stirred for 60 min. The homogeneous solution was then transferred to a 100 mL Teflon-lined autoclave and subjected to a solvothermal reaction at 160 °C for 3 h. The obtained products were washed three times with ethanol and deionized water, centrifuged, filtered, and dried at 60 °C for later use. BiOBr samples prepared using different solvents without the addition of PVP were named BiOBr-Me, BiOBr-EG, BiOBr-GI, or BiOBr-Ma. BiOBr samples prepared with different solvents added to PVP were named BiOBr-Me-PVP, BiOBr-EG-PVP, BiOBr-GI-PVP, or BiOBr-Ma-PVP, respectively. For control purposes, BiOBr samples synthesized using water as the solvent were labeled as BOB-W (without PVP) or BOB-P (with PVP). Additionally, BiOBr samples prepared with ethylene glycol as the solvent and varying amounts of PVP (0.4 g, 0.6 g, and 0.8 g) were referred to as BOB-0.4, BOB-0.6, or BOB-0.8, respectively. 

### 3.3. Characterization of Photocatalysts

The crystal structure of the samples was evaluated by X-ray powder diffraction (XRD, Rigaku Ultima IV, Rigaku Corporation, Tokyo, Japan). The functional groups were identified by Fourier transform infrared spectroscopy (FTIR, Thermo Fisher Scientific Nicolet iS20, Thermo Fisher Scientific, Waltham, MA, USA) in the range 500–4000 cm^−1^. The surface morphology and elemental compositions of the samples were analyzed by scanning electron microscopy (SEM, ZEISS GeminiSEM 300, Carl Zeiss AG, Oberkochen, Germany) and transmission electron microscopy (TEM, JEOL JEM F200, JEOL Ltd., Tokyo, Japan) coupled with Energy Dispersive X-ray spectroscopy (EDS). The specific area and pore characteristics were measured by the Brunauer-Emmett-Teller method (BET, Micromeritics ASAP 2460, Micromeritics Instrument Corporation, Norcross, GA, USA). Surface electronic states were recorded by X-ray photoelectron spectroscopy (XPS, Thermo Scientific ESCALAB 250Xi, Thermo Fisher Scientific, East Grinstead, UK). The UV-vis diffusive reflectance spectra (DRS) of samples were analyzed on a UV-vis spectrophotometer (Shimadzu UV-3600i Plus, Shimadzu Corporation, Kyoto, Japan). Electrochemical impedance spectroscopy (EIS) and Mott-Schottky measurements were carried out using a three-electrode system of a CHI760E electrochemical workstation (CH Instruments, Inc., Austin, TX, USA). Electron paramagnetic resonance (EPR) spectra were collected on Bruker EMXnano EPR spectrometer (Bruker Corporation, Billerica, MA, USA).

### 3.4. Photodegradation Measurements

The photodegradation experiments were conducted using a 300 W Xe lamp (λ > 420 nm) to evaluate the degradation performance of MO, RhB, and CIP. In detail, 20 to 70 mg of photocatalyst was added to 50 mL of pollutant solution (20 mg·L^−1^). The mixture was first stirred in the dark for 30 min to achieve adsorption–desorption equilibrium, followed by photocatalytic degradation until the pollutants were nearly completely removed. At predetermined intervals, samples were collected, filtered through a 0.45 μm membrane, and analyzed using a UV-visible (UV-vis) spectrophotometer (TU1810, Puxi Co., Beijing, China) to monitor the degradation of MO, RhB, and CIP at wavelengths of 466 nm, 554 nm, and 276 nm, respectively. To assess the stability and practical applicability of the catalyst, experiments were performed under varying conditions, including changes in catalyst dosage, initial pH (adjusted with 1 mol/L of NaOH and HNO_3_), and the addition of inorganic anions (1 mM Cl^−^, HCO_3_^−^, SO_4_^2−^, PO_4_^3−^, Al^3+^, and Ca^2+^ into the RhB solution), and HA (10 mg/L) to simulate the coexistence of organic pollutants. To identify the active species involved in the photocatalytic reaction, 1.0 mM BQ, 5 mL IPA and 1.0 mM EDTA-2Na were added as •O_2_^−^, •OH, and h^+^ quenchers, respectively [60,61].

### 3.5. DFT Calculation

Density functional theory (DFT) was implemented in the PWmat package-20240223 leveraging GPU [62,63]. PWmat is used to calculate the electrical and structural properties, as well as the defect properties, of the three structures. To optimize the geometry for the exchange-correlation potential, the generalized gradient approximations (GGA) [64,65] of the Perdew-Burke-Ernzerhof (PBE) functional [66] were utilized, with the convergence criteria being a force tolerance of 0.01 eV/Å. Also employed for band structure and density of states was the Heyd-Scuseria-Ernzerhof (HSE06) function [67], which has a Fock exchange parameter of *ω* = 0.3 and a screening parameter [68] of *ω* = 0.2 for HSE-like hybrid functionals. Meshes of 0.04/Å were used for geometry optimization in the Monkhorst-Pack k-points. Norm-Conserving Pseudopotential [69,70] with a cutoff energy of 60 Rydberg was used for all the calculations in the PWmat package.

## 4. Conclusions

The synergism between the addition of PVP and the use of hydroxyl-rich solvents in the solvothermal process of preparing BiOBr has been revealed for the first time. The utilization of hydroxyl-rich solvents significantly increased the surface hydroxyl group density of BiOBr, enhancing its adsorption affinity of organic pollutants. Concurrently, the introduction of PVP not only guided the formation of hierarchical microspheres with an expanded specific surface area and optimized pore architecture but also synergistically amplified surface hydroxylation. Crucially, the incorporation of PVP induced a positive shift in the valence band (VB) position of BiOBr-EG-PVP from 2.22 eV to 2.36 eV, substantially boosting its photooxidative capacity by enhancing h^+^-mediated oxidation kinetics. This dual-functional strategy, i.e., simultaneously engineering a microstructure and electronic properties through hydroxyl group modulation and surfactant templating, provides a universal framework for designing advanced photocatalysts. This study offered a method for synergistically optimizing the microstructure and band structure of BiOBr using hydroxyl groups and surfactants, which might also be applied to the enhanced photooxidative ability of other novel photocatalysts.

## Data Availability

Data are contained within the article and Supplementary Materials.

## Supplementary Materials

The following supporting information can be downloaded at: https://www.mdpi.com/article/10.3390/molecules30061286/s1, Figure S1: XRD patterns of BiOBr with different PVP amount addition; Figure S2: SEM images of (a) BiOBr-Me, (b) BiOBr-Me-PVP, (c) BiOBr-EG, (d) BiOBr-EG-PVP, (e) BiOBr-Gl, (f) BiOBr-Gl-PVP, (g) BiOBr-Ma, (h) BiOBr-Ma-PVP under low magnification factor; Figure S3: SEM images of BOB-W and BOB-P; Figure S4: SEM images of BOB-0.8; Figure S5: XPS spectra of O 1s (BiOBr-Me, BiOBr-Me-PVP, BiOBr-EG,BiOBr-EG-PVP, BiOBr-Gl, BiOBr-Gl-PVP, BiOBr-Ma, BiOBr-Ma-PVP); Figure S6:Photodegradation of RhB with BiOBr-EG-PVP and the first-order-kinetic plots and corresponding apparent rate constants k of BiOBr-EG-PVP under different dose; Figure S7: Stability of BiOBr-EG-PVP photocatalyst at different pH levels; Table S1: BET surface areas pore distribution and of prepared BiOBr catalysts; Table S2: The hydroxyl and lattice oxygen content of prepared BiOBr catalysts; Table S3: First-order Kinetic Constants (k) and relative coefficients (R^2^) of prepared BiOBr with different given dosage; Table S4: First-order Kinetic Constants (k) and relative coefficients (R^2^) of prepared BiOBr.
