# Supplementary material for: Synergistic Mechanism of Hydroxyl Regulation and a Polyvinylpyrrolidone Surfactant in Enhancing the Catalytic Oxidation Abilities of BiOBr
Source: Molecules. 2025 Mar 13;30(6):1286. doi: 10.3390/molecules30061286 (PMC11945476; doi:10.3390/molecules30061286)
Supplement: Supplementary file 1 [file molecules-30-01286-s001.zip › molecules-3514385-supplementary.pdf]

Supporting Information

Synergistic Mechanism of Hydroxyl Regulation and a Polyvinylpyrrolidone Surfactant in Enhancing the Catalytic Oxidation Abilities of BiOBr

Yiran Zhang [1], Boyuan Xuan [1], Jiekai Wang [1], Xiang Chen [2], Changwei Zhao [1,] *, Lixia Zhao [3] and Jing Kang [4]

[1] Beijing Key Laboratory of Farmland Soil Pollution Prevention and Remediation, College of Resources and Environmental Sciences, China Agricultural University, Beijing, 100193, China
[2] State Key Laboratory of Water Environment Simulation, School of Environment, Beijing Normal University, Beijing 100875, China
[3] State Key Laboratory of Environmental chemistry and Ec-Toxicology, Research Center for Eco-Envronmental sciences, Chinese Academy of sciences, Beijing 100085, China
[4] China Institute for Radiation Protection, Taiyuan 030006, China
* Correspondence: zhaocw@cau.edu.cn; Tel.:+86 15210835956

Tables:

Table S1. BET surface areas pore distribution and of prepared BiOBr catalysts.

Treatments	BET surface area (m^2/g)	Pore volume (cm^3/g)	Micropore volume (cm^3/g)	Average pore diameter (nm)
BiOBr-Me	18.05	0.12	0.0026	27.05
BiOBr-EG	18.38	0.057	0.0011	12.36
BiOBr-Gl	3.05	0.021	0.00064	27.69
BiOBr-Ma	6.16	0.027	0.0020	17.33
BiOBr-Me-PVP	13.28	0.051	0.00015	15.36
BiOBr-EG-PVP	24.87	0.079	0.00051	12.66
BiOBr-Gl-PVP	4.15	0.028	0.0018	27.31
BiOBr-Ma-PVP	20.70	0.079	0.0011	15.23
BOB-W	2.55	0.021	0.00077	32.42
BOB-P	11.29	0.061	0.00015	20.82
BOB-0.4	14.00	0.0017	0.0017	30.63
BOB-0.6	14.51	0.0013	0.0013	23.57
BOB-0.8	9.48	0.0027	0.0027	28.01

Table S2. The hydroxyl and lattice oxygen content of prepared BiOBr catalysts.

Samples	Hydroxyl groups (%)	Lattice oxygen (%)	Bi:O:Br
BOB-W	0	100	1:2.11:1.18
BiOBr-Me	23.08	76.92	1:1.33:1.15
BiOBr-Me-PVP	58.82	41.18	1:1.85:1.18
BiOBr-EG	47.37	52.63	1:1.56:1.05
BiOBr-EG-PVP	55.25	44.75	1:1.92:1
BiOBr-Gl	31.97	68.03	1.36:3.50:1
BiOBr-Gl-PVP	9.09	90.91	1.54:5.10:1
BiOBr-Ma	32.89	67.11	1:1.47:1.14
BiOBr-Ma-PVP	70.92	29.08	1:2.21:1.21
BOB-0.4	69.47	30.53	1:2.16:1.10
BOB-0.6	70.76	29.24	1:2.13:1.08
BOB-0.8	68.59	31.41	1:2.10:1.07

Table S3. First-order Kinetic Constants (k) and relative coefficients (R^2) of prepared BiOBr with different given dosage.

Given dosage (g/L)	Kinetic Constants k (min^{-1})	Relative coefficients R^2
0	0.00405	0.93626
0.4	0.11816	0.97691
0.6	0.19358	0.94154
0.8	0.23246	0.974922
1.0	0.28460	0.97447
1.2	0.28266	0.95174
1.4	0.25689	0.98940

Table S4. First-order Kinetic Constants (k) and relative coefficients (R^2) of prepared BiOBr.

Samples	Kinetic Constants k (min^{-1})			Relative coefficients R^2		
	CIP	RhB	MO	CIP	RhB	MO
BOB-W	0.00538	0.00641	0.00122	0.91953	0.95656	0.90378
BOB-P	0.01266	0.13773	0.00938	0.99545	0.91852	0.99552
BiOBr-Me	0.04961	0.15247	0.00588	0.90996	0.99163	0.98725
BiOBr-Me-PVP	0.4754	0.18029	0.00336	0.90668	0.90918	0.99699
BiOBr-EG	0.03975	0.09287	0.00321	0.99817	0.96717	0.90224
BiOBr-EG-PVP	0.54732	0.28460	0.01605	0.96087	0.97447	0.97308
BiOBr-Gl	0.00862	0.07283	0.0183	0.94486	0.92313	0.99396
BiOBr-Gl-PVP	0.01241	0.07549	0.03608	0.97877	0.90199	0.9778
BiOBr-Ma	0.02982	0.12639	0.00269	0.97262	0.99209	0.93876
BiOBr-Ma-PVP	0.01837	0.15362	0.00695	0.94007	0.90834	0.99329

Figures:

Figure S1. XRD patterns of BiOBr with different PVP amount addition.

Figure S2. SEM images of (a) BiOBr-Me, (b) BiOBr-Me-PVP, (c) BiOBr-EG, (d) BiOBr-EG-PVP, (e) BiOBr-Gl, (f) BiOBr-Gl-PVP, (g) BiOBr-Ma, (h) BiOBr-Ma-PVP under low magnification factor.

Figure S3. SEM images of BOB-W and BOB-P.

Figure S4. SEM images of BOB-0.8.

Figure S5. XPS spectra of O 1s (BiOBr−Me, BiOBr−Me−PVP, BiOBr−EG,BiOBr−EG−PVP, BiOBr−Gl, BiOBr−Gl−PVP, BiOBr−Ma, BiOBr−Ma−PVP).

Figure S6. Photodegradation of RhB with BiOBr-EG-PVP and the first-order-kinetic plots and corresponding apparent rate constants *k* of BiOBr-EG-PVP under different dose.

Figure S7. Stability of BiOBr-EG-PVP photocatalyst at different pH levels.

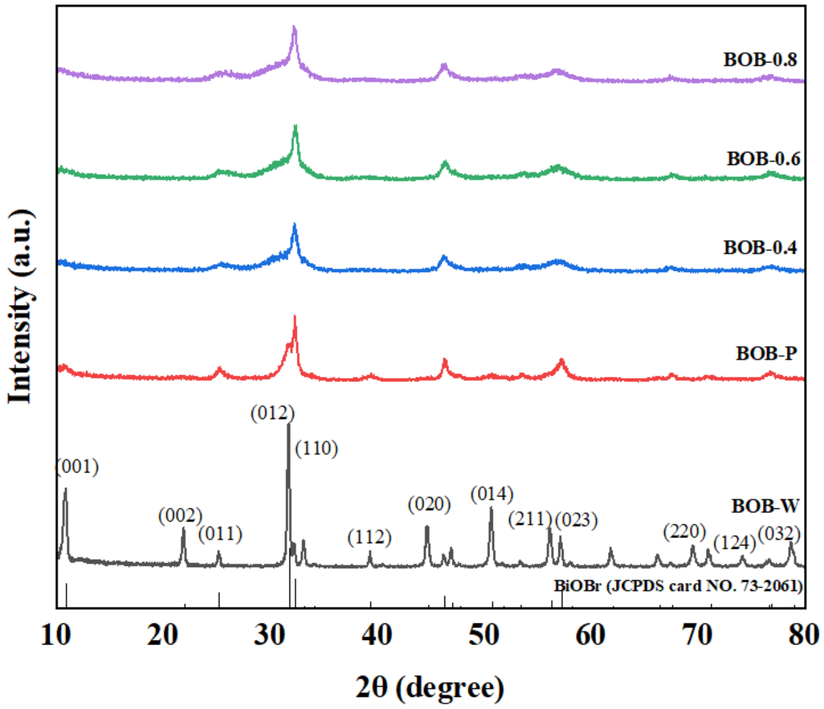

Figure S1. XRD patterns of BiOBr with different PVP amount addition.

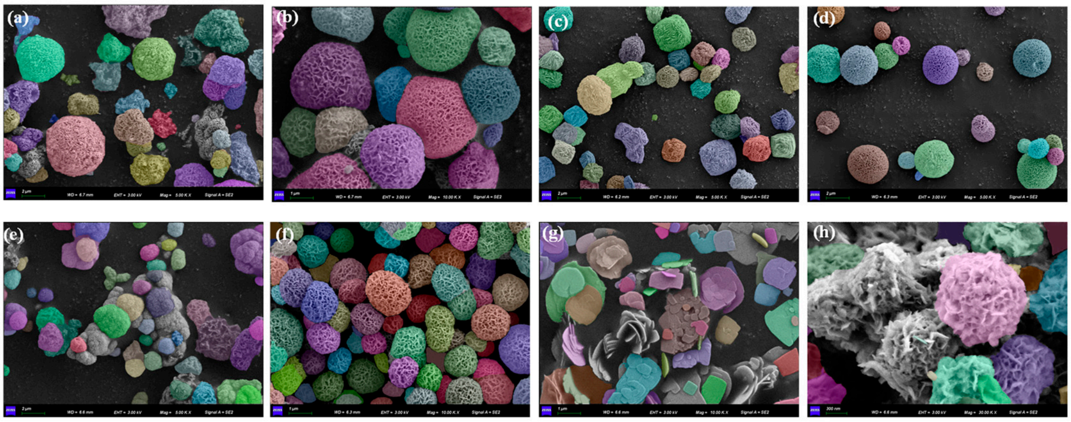

Figure S2. SEM images of (a) BiOBr-Me, (b) BiOBr-Me-PVP, (c) BiOBr-EG, (d) BiOBr-EG-PVP, (e) BiOBr-Gl, (f) BiOBr-Gl-PVP, (g) BiOBr-Ma, (h) BiOBr-Ma-PVP under low magnification factor.

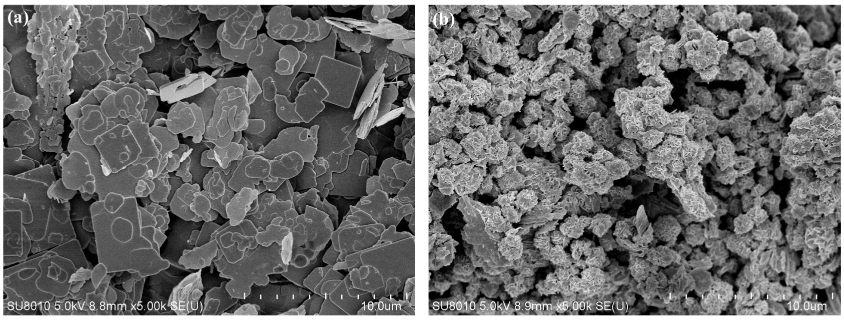

Figure S3. SEM images of (a) BOB-W and (b) BOB-P.

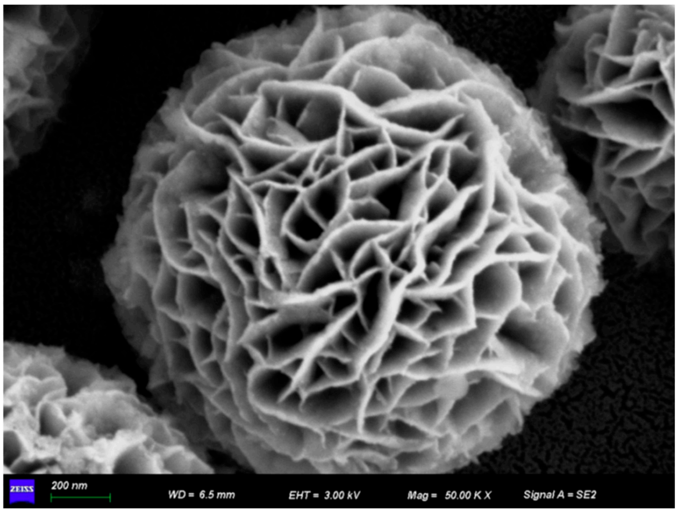

Figure S4. SEM images of BOB-0.8.

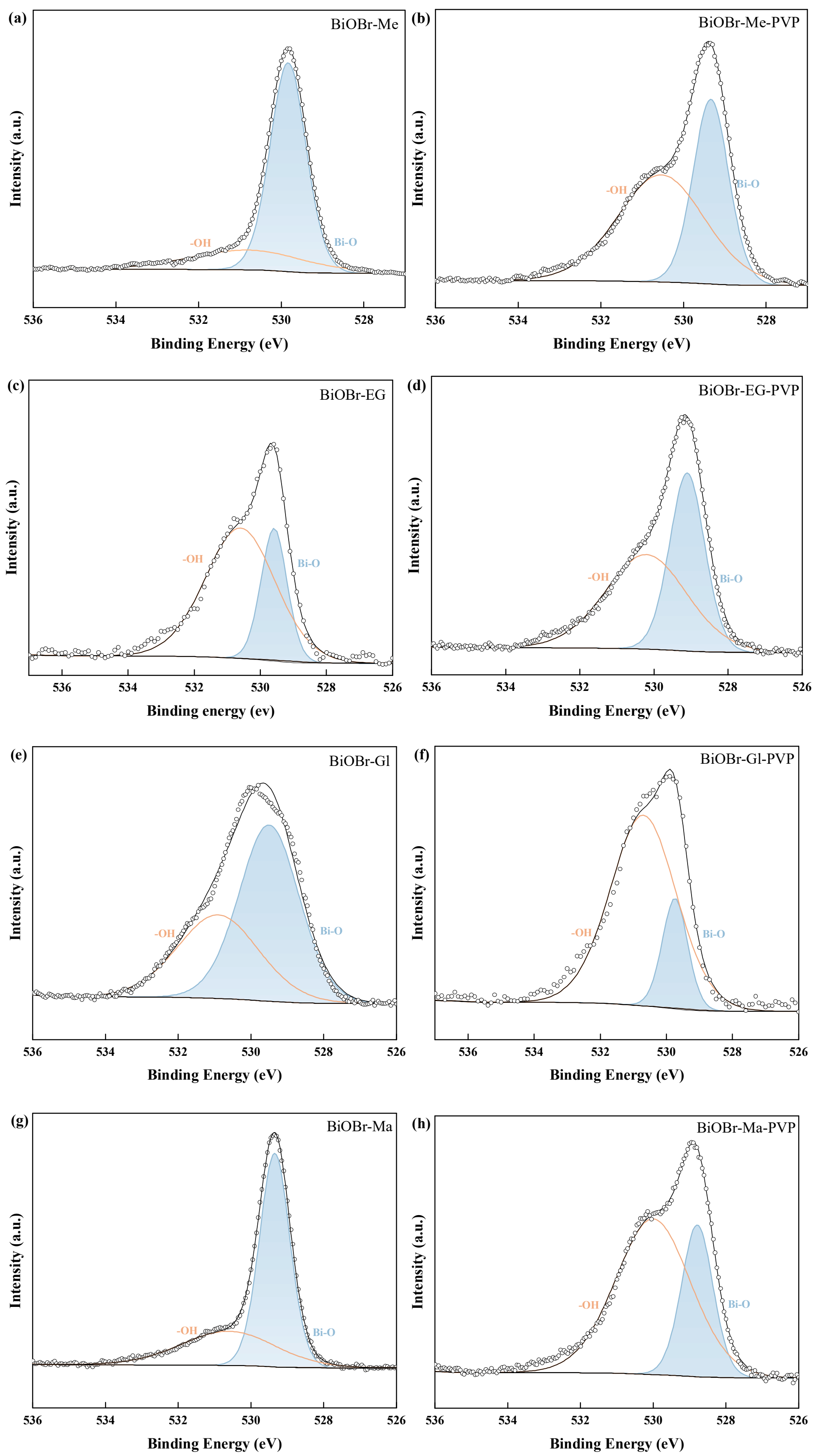

Figure S5. XPS spectra of O 1s (a) BiOBr−Me, (b) BiOBr−Me−PVP, (c) BiOBr−EG, (d) BiOBr−EG−PVP, (e) BiOBr−Gl, (f) BiOBr−Gl−PVP, (g) BiOBr−Ma, and (h) BiOBr−Ma−PVP.

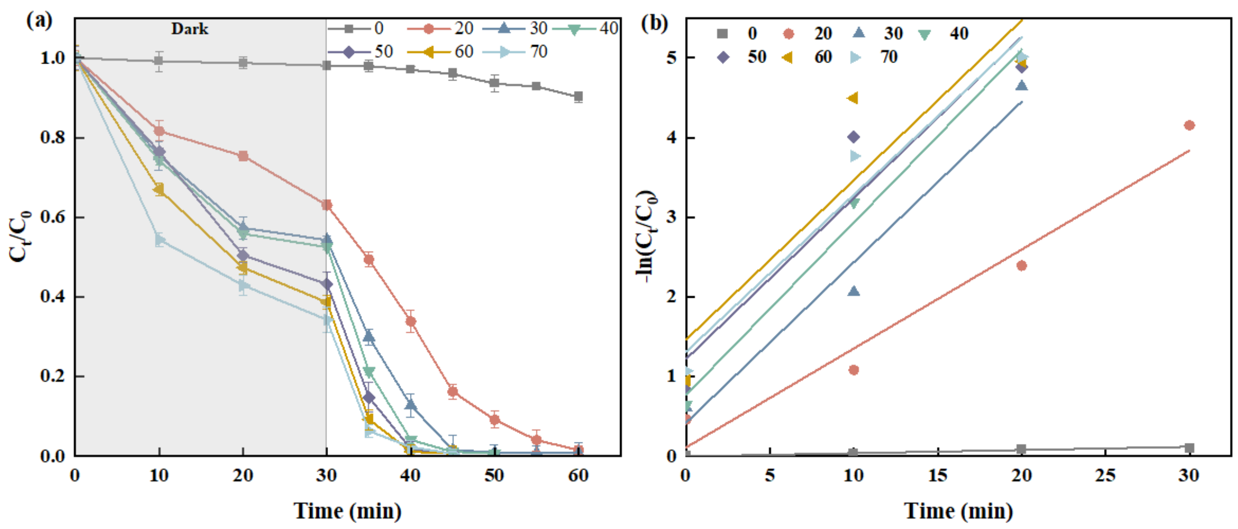

Figure S6. (a) Photodegradation of RhB with BiOBr-EG-PVP under different dose and (b) The first-order-kinetic plots and corresponding apparent rate constants *k* of BiOBr-EG-PVP under different dose.

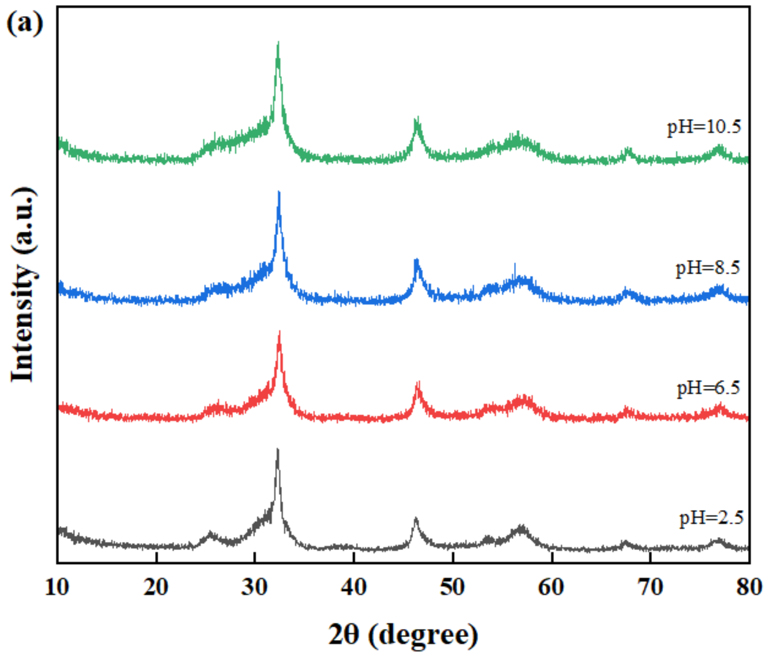

Figure S7. Stability of BiOBr-EG-PVP photocatalyst at different pH levels.
